# Retrospective audit compares screening and treatment of pregnancy-related anaemia in regional New South Wales with Australian guidelines

**DOI:** 10.1186/s12884-024-06634-5

**Published:** 2024-07-03

**Authors:** Mariam Ebrahim, Priya Dharshini Vadive, Tegan Dutton, Anayochukwu Edward Anyasodor, Uchechukwu Levi Osuagwu, Jannine Bailey

**Affiliations:** 1 School of Medicine, Bathurst Rural Clinical School (BRCS), Western Sydney University, PO Box 9008, Bathurst, NSW 2795 Australia; 2 Rural Health Research Institute, Charles Sturt University, Orange, NSW 2800 Australia

**Keywords:** Pregnancy, Anaemia, Guidelines, Iron supplements, Rural

## Abstract

**Background:**

Anaemia during pregnancy is common worldwide. In Australia, approximately 17% of non-pregnant women of reproductive age have anaemia, increasing to a rate of 25% in pregnant women. This study sought to determine the rate of screening for anaemia in pregnancy in regional New South Wales, and to determine whether screening and treatment protocols followed the recommended guidelines.

**Methods:**

This retrospective study reviewed antenatal and postnatal (48 h) data of women (*n* = 150) who had a live birth at Bathurst Hospital between 01/01/2020 and 30/04/2020. Demographic data, risk factors for anaemia in pregnancy, antenatal bloods, treatments provided in trimesters one (T1), two (T2) and three (T3), and postpartum complications were recorded. These were compared to the Australian Red Cross Guidelines (ARCG) using descriptive statistics.

**Results:**

Of the women with screening data available (*n* = 103), they were mostly aged 20-35yrs (79.6%), 23.3% were obese, 97.1% were iron deficient, 17% were anaemic and only a few (5.3%) completed the full pregnancy screening as recommended by the ARCG while a majority completed only partial screenings specifically Hb levels in T1 (56.7%), T2 (44.7%) and T3 (36.6%). Compliance to oral iron was largely undocumented, but constipation was a common side effect among the women. IV iron was administered in 14.0% of women, approximately 1.75x higher than the recommended rate.

**Conclusions:**

This study provided useful information about compliance to screening and treatment guidelines for anaemia in pregnancy. We identified the need for improved documentation and communication between various health providers to ensure adequate antenatal care to prevent maternal complications during pregnancy. This will improve patient care and encourage further developments in maternal care, bridging the rural health gap.

**Supplementary Information:**

The online version contains supplementary material available at 10.1186/s12884-024-06634-5.

## Introduction

Anaemia, which is primarily caused by iron deficiency is a significant health issue in pregnancy worldwide [1], as it affects nearly 50% of pregnant women [2]. Untreated pregnancy-related anaemia is associated with adverse foetal outcomes such as preterm birth, low still birth and perinatal death [1–3]. Its impact on the neurodevelopment of children has also been reported [4]. Adjunct to the effects of anaemia in pregnancy is maternal morbidity, including impaired quality of life, need for transfusion therapy, post-partum haemorrhage and maternal mortality [2, 5]. The global prevalence of anaemia in pregnancy is estimated at 37% [1, 6]. In Australia, 15.7% of pregnancies are reported to be affected by anaemia [6], and this rate increases with rurality [7]. However, data lack to rationalise anaemia prevalence, screening and treatment in Bathurst, New South Wales (NSW).

The average dietary iron deficiency for pregnant women in Australia is 27 mg/day, and it is known that there is a threefold increase in the physiological demand for iron, which necessitates a total of 1000–1200 mg throughout the gestation period [8]. This increase in physiological demand for iron occurs in the second trimester [9, 10], and peaks in the third trimester with various physiological processes [11]. These processes involve the expansion of erythrocytic mass, growth of maternal tissues, as well as foetal and placental development [12]. In addition, iron functions as a reserve to compensate for potential blood loss during birth [13].

Despite the recommendation of more than one antenatal visit during pregnancy, the Australian Institute of Health and Welfare reported in 2020 that the frequency of antenatal visits varied by remoteness, with a record of 95% attendance of 5 or more generally and 92% attending 5 or more in remote areas [14]. The preceding highlights the importance of anaemia screening, which informs available treatment options to ensure the well-being of pregnant women and optimal options that favour both the expectant mother and the developing foetus.

Since iron cannot be synthesised by the body, individuals with iron deficiency anaemia require iron supplementation, either through oral or intravenous administration to ensure adequate supply to the body [15]. It has been shown that the mentioned options offer effective treatment for iron deficiency [16], but there is a suggestion that oral replacement is recommended as first line of treatment [17]; and usually taken with vitamin C to optimise iron absorption [18]. Interestingly, intravenous administration exhibits a better efficacy in improving maternal haematological indices such as haemoglobin [18] and ferritin [19] levels, and producing immediate outcomes. Nevertheless, due to reported gastrointestinal adverse effects, adherence to oral iron supplementation is reducing, resulting to resurgence in the use of intravenous iron [20].

In Australia, clinical guidelines are available for the screening and treatment of anaemia in pregnancy [8, 21], but this study was focused on the guidelines of the Australian Red Cross 2020 Antenatal Audit tool (hereafter referred to as the Red Cross Guidelines [ARCG]). Due to the limited data on the prevalence, screening, and treatment of anaemia in regional and rural Australian population, this study was designed to compare the screening and treatment of pregnancy-related anaemia in a regional Australian hospital based on the ARCG.

## Methods

### Study design and setting

This is a retrospective study conducted at Bathurst Hospital (BH), involving audit of hospital records of pregnant women for data on anaemia in pregnancy. The BH is part of Bathurst Health Service, which provides services to Bathurst residents and its neighbours. The health institution is a rural hospital that functions as a training facility for medical students at Western Sydney University and Charles Sturt University [22]. The hospital facilitates approximately 500 births per year [23], and the antenatal clinic is supervised by a Consultant paediatrician assisted by trained registered nurses.

### Model of care

The Bathurst Maternity Health Service offers women with access to GP obstetric care from three trained practitioners for comprehensive support throughout pregnancy. This ensures timely assessment by a midwife before 20 weeks for seamless birth preparation and personalised care. Women can also receive specialised care and second opinions from obstetricians, who also serve in the hospital’s maternity unit. The models of care for maternity care include Midwifery Led Antenatal Clinic (MLAC) which offers personalised pregnancy care with midwives, who may refer patients to an obstetrician if needed. There is a midwifery group practice (MGP) that offers continuity of care with a dedicated midwife, offering individualised support from the antenatal to the postnatal period. Also, the Aboriginal Maternal Infant Health Service which is delivered by Aboriginal Health Workers and midwives provides tailored care for Indigenous mothers and babies.

### Study population

The study enrolled all women, who gave birth at BH between the dates of 1st January 2020 and 30th April 2020. Due to the time required to go through all the hospital records (power chart and e-maternity) for data extraction, data collection was limited to three months, and the choice of study period was randomly chosen by the supervisory team. Despite the COVID-19 pandemic, at the time of this study, all appointments to the clinic were conducted in person with minimal change to standard procedures. The study did not involve COVID-19 patients, due to the need to minimise the spread of the infection. Furthermore, considering the relationship between COVID-19 and anaemia, data for patients with COVID-19 were excluded from the study. There were some missing data for women who either did not complete the test had no record of the test in the system, paper notes, or women who completed the test through another provider (e.g., a general practitioner). However, all the needed information available in their hospital record was documented and the data collection tool was modified in line with the RCMG.

### Definition of red cross maternity guidelines

The ARCG recommends screening for anaemia for all women in the first and second trimesters. However, third-trimester screening is only recommended to monitor for women, who were previously iron deficient; and had been prescribed an oral iron supplement. Documentation for the risk factor for anaemia is ensured within the first-trimester visit, and preliminary Full Blood Counts (FBC) as well as iron studies completed. Participants with low haemoglobin (Hb) (< 110 g/L) and low ferritin (≤ 30 mcg/L), or exclusively low ferritin are recommended an oral iron supplement, the dose-dependent on the severity of their deficiencies. Subsequently, women who are noted as having severe anaemia, or abnormalities in their blood are referred to a specialist.

Within the second trimester, repeat Hb and ferritin levels are conducted. All participants previously on oral iron are recommended to continue taking the supplement. Participants with low Hb (< 105 g/L) and/or low ferritin (≤ 30 mcg/L), a ≥ 15 g/L fall in Hb (compared to the first trimester), or a high risk of postpartum haemorrhage (PPH, i.e. the loss of 500 mL of blood or more after childbirth [24]) are additionally recommended as an oral iron supplement. In the third trimester, repeat Hb and ferritin levels are tested to assess response to oral iron. All women are advised to continue taking the previously prescribed oral iron. If Hb and ferritin levels are not sufficiently responsive to the oral iron supplements or participants have failed to regularly take the iron, due to intolerance or non-compliance, intravenous iron is indicated. This explanation has been demonstrated in the figures detailing the recommendations for first, second and third trimester (see Appendix A1-3, respectively).

### Ethical consideration

Ethics approval for this study was sought and granted by the Greater Western Human Research Ethics Committee in 2020 (ETH00244; expiration date: February 2026); and the Western Sydney University Human Research Ethics Committee (H14327). To ensure participant confidentiality, all data were stored on a password-protected database and de-identified. All participants gave informed consent before enrolment.

### Data collection

Participants’ information was extracted from their medical records available in e-maternity and Power chart. This was undertaken by a trained medical student and was supervised by a midwife and the research team. Data collected included medical record number (MRN), sociodemographic factors, risk factors, general medical history, obstetric history, antenatal blood, prescription of iron (oral or intravenous), and birth complications (see Appendix B for a detailed list of variables). To maintain anonymity, data were transferred securely to a password-protected database where participants were de-identified (removal of MRN and birth date), upon completion of data collection.

Data were categorised into various timelines, based on the ARCG [25], which requires documentation of risk factors for anaemia, intrapartum, and postnatal (12–24 h post birth) to record completed blood tests, each time block was given a +/- 2 weeks leeway (e.g., blood taken at 24 weeks were included in the second trimester). In instances involving the completion of more than one blood test within one timeframe (indicated above), the first complete set of blood was prioritised. Where different parts of the blood tests were completed separately, results were combined, on the basis that they both fitted into the relevant timeframes. The guidelines require that a full blood count and ferritin should be requested in the first trimester (T1: >20 weeks gestation), and in the second trimester (T2: 26–28 weeks gestation), a full blood count and ferritin is recommended, while in the third trimester (T3: 32–36 weeks gestation), repeat full blood count and ferritin is required.

### Data analysis

Descriptive statistics were performed using Jamovi software, version 2.3 (Jamovi, Sydney, Australia). The proportion screened for anaemia, those with and without iron deficiency, those with anaemia, and those who were treated with prescribed oral, or intravenous iron supplementation were calculated, and comparisons were made against the ARCG to assess for concordance to screening and treatment protocols.

## Results

### Demographics of the study population

Table 1 displays the demographic characteristics of the study population. Out of 150 mothers who gave birth during the 4-month study period, 11.7% identified as Aboriginal or Torres Strait Islanders. Over half of the pregnancies were planned, with or without assisted reproductive technologies. The majority (93.2%) reported no dietary restrictions. Most mothers were aged between 20 and 35 years old (79.6%). Iron deficiency was prevalent, with most participants affected, and 17% of these cases met the criteria for iron deficiency anaemia.

**Table 1:** Rates of iron deficiency based on Red Cross Guidelines, divided by participant risk factors

Variables	Total (*n* = 103)	Iron Deficient*	Iron Deficiency Anaemia*	Iron (oral or IV) recommended *	Not iron deficient*
**Demography**					
**Age group (years)**					
<20	4 (3.9%)	4 (3.9%)	0 (0.0%)	4 (3.9%)	0 (0.0%)
20–35	82 (79.6%)	65 (63.1%)	15 (14.6%)	80 (77.7%)	2 (1.9%)
>35	17 (16.5%)	14 (13.6%)	2 (1.9%)	16 (15.5%)	1 (1.0%)
**Nationality**					
ATSI**	12 (11.7%)	8 (7.8%)	4 (3.9%)	12 (11.7%)	0 (0.0%)
Other	91 (88.3%)	75 (72.8%)	13 (12.6%)	88 (85.4%)	3 (2.9%)
**Behavioural Factors**					
**Diet**					
No restrictions	96 (93.2%)	77 (74.8%)	16 (15.5%)	93 (90.3%)	3 (2.9%)
Vegetarian/vegan	7 (6.8%)	6 (5.8%)	1 (1.0%)	7 (6.8%)	0 (0.0%)
**Weight**					
Obesity	24 (23.3%)	22 (21.4)	1 (1.0%)	23 (22.3%)	1 (1.0%)
Normal Weight	78 (75.7%)	60 (58.3%)	16 (15.5%)	76 (73.8%)	2 (1.9%)
Unknown Weight	1 (1.0%)	1 (1.0%)	0 (0.0%)	1 (1.0%)	0 (0.0%)
**Medical History**					
**History of Anaemia**					
Yes	31 (30.1%)	23 (22.3%)	8 (7.7%)	31 (30.1%)	0 (0.0%)
No	72 (69.9%)	60 (58.3%)	9 (8.7%)	69 (67.0%)	3 (2.9%)
**Recent significant History of Bleeding**					
Yes	1 (1.0%)	1 (1.0%)	0 (0.0%)	1 (1.0%)	0 (0.0%)
No	102 (99.0%)	82 (79.6%)	17 (16.5%)	99 (96.1%)	3 (2.9%)
**Obstetric History**					
**Parity**					
≥ 3	68 (66.0%)	51 (49.5%)	14 (13.6%)	65 (63.1%)	3 (2.9%)
<3	35 (34.0%)	32 (31.1%)	3 (3.9%)	35 (34.0%)	0 (0.0%)
**Interpregnancy Interval**					
< 1 year	18 (17.5%)	16 (15.5%)	1 (1.0%)	17 (16.5%)	1 (1.0%)
≥ 1 year	85 (82.5%)	67 (65.0%)	16 (15.5%)	83 (80.6%)	2 (1.9%)
**PPH**					
Previous PPH	13 (12.6%)	13 (12.6%)	0 (0.0%)	13 (12.6%)	0 (0.0%)
No previous PPH	90 (87.4%)	70 (68.0%)	17 (16.5%)	87 (84.5%)	3 (2.9%)
**Antenatal History**					
**Conception**					
Planned ***	67 (65.0%)	59 (57.3%)	7 (6.8%)	66 (64.1%)	1 (1.0%)
Unplanned	36 (35.0%)	24 (23.3%)	10 (9.7%)	34 (33.0%)	2 (1.9%)
**Antenatal Care**					
Hospital Care	67 (65.0%)	54 (52.4%)	11 (10.7%)	65 (63.1%)	2 (1.9%)
GP Care	15 (14.6%)	13 (12.6%)	2 (1.9%)	15 (14.6%)	0 (0.0%)
Shared Care	21 (20.4%)	16 (15.5%)	4 (3.9%)	20 (19.4%)	1 (1.0%)
**Total Participants**	103 (100%)	83 (80.6%)	17 (16.5%)	100 (97.1%)	3 (2.9%)

* In reference to Red Cross maternity guidelines

** Aboriginal and Torres Strait Islander

*** Planned refers to natural and Assisted Reproductive Technology (ART) conceptions

### Screening for anaemia in pregnancy by trimester

Table 2 presents the screening rates by level of completion in each trimester. Few women completed the recommended screenings in trimesters one (29.3%) and three (47.9%), the majority had partial screening, especially for Hb levels across the three trimesters (T1: 56.7%, T2: 44.7%, T3: 36.6%) and many women had no screening, mostly in their second trimester (24.7%). Across the three trimesters, women in this hospital were either not/or seldomly screened for iron levels during pregnancy.

**Table 2:** Rates of screening completion based on trimesters

Trimester	Full screening	Partial Screening**		No screening recorded
		**Hb only**	**Ferritin only**	
First, *n* = 150	44 (29.3%)	85 (56.7%)	3 (2.0%)	18 (12.0%)
Second, *n* = 150	46 (30.7%)	67 (44.7%)	0 (0.0%)	37 (24.7%)
Third, *n* = 71 *	34 (47.9%)	25 (35.2%)	1 (1.4%)	11 (15.5)

*3rd−trimester screening is recommended based on previous screening results, thus according to the guidelines, screening could not be recommended for participants where there was insufficient data recorded for Trimester 1 and Trimester 2. **participants had either haemoglobin or ferritin values available

**Fig. 1:**
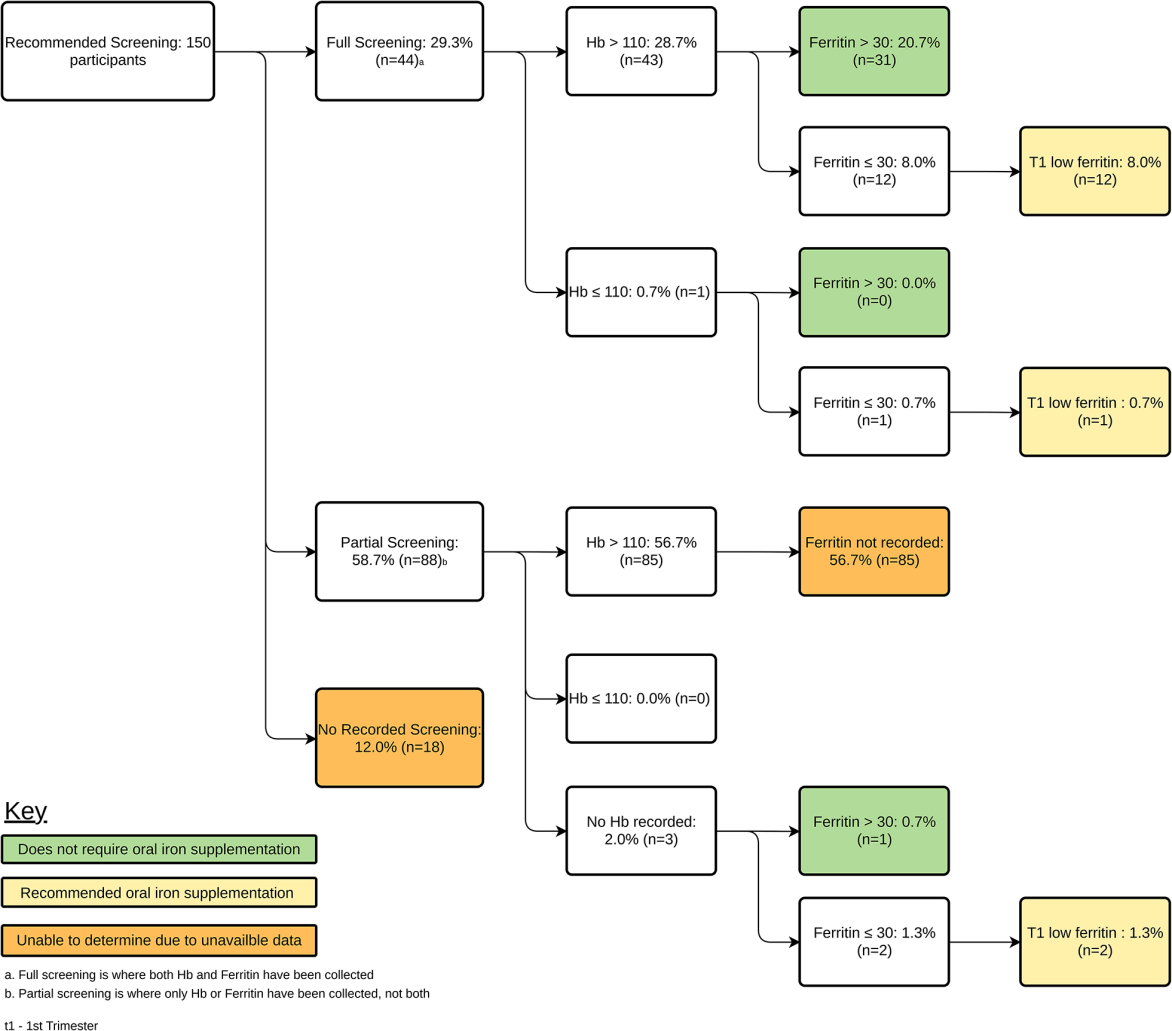
Rates of screening and iron deficiency in the first trimester

**Fig. 2:**
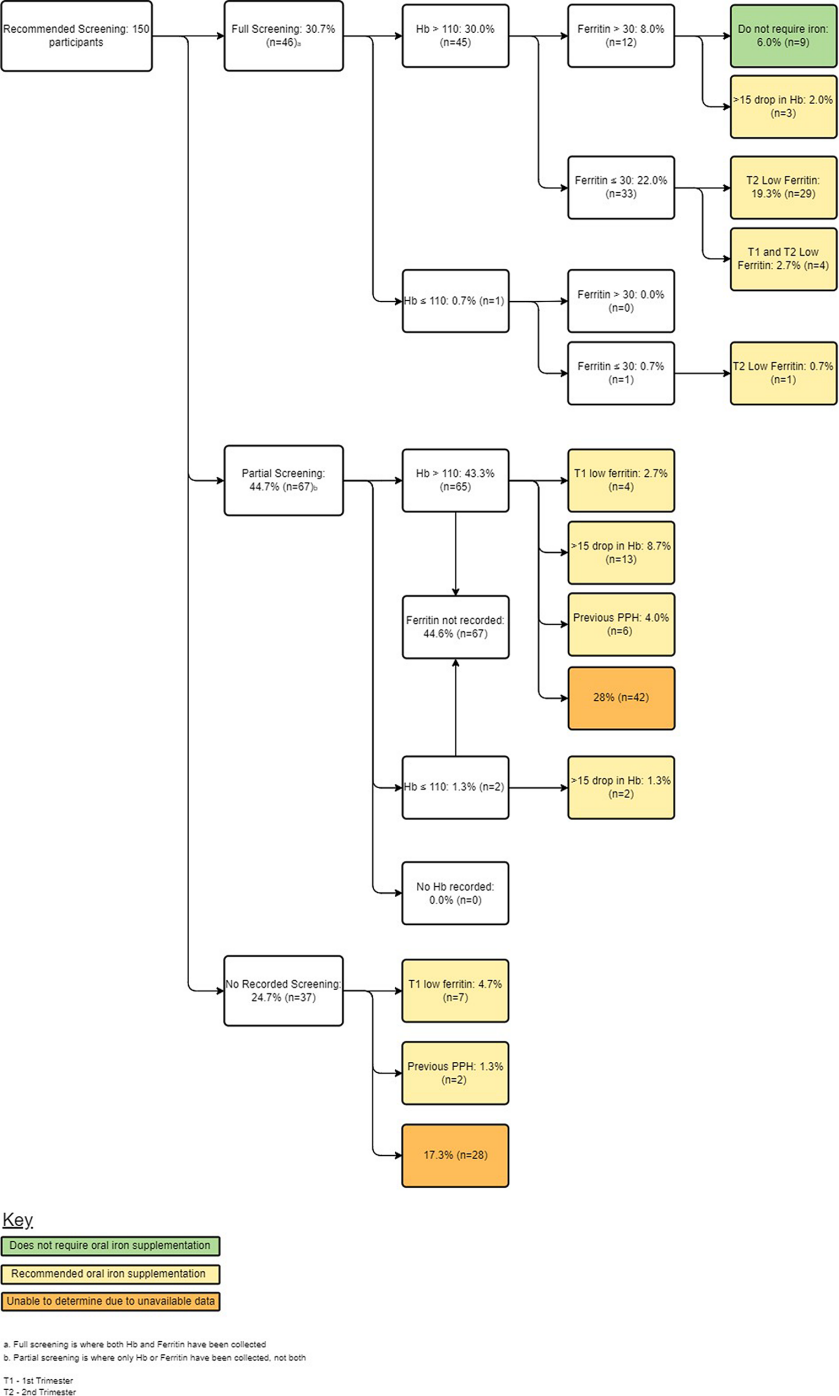
Rates of screening and iron deficiency in the second trimester

**Fig. 3:**
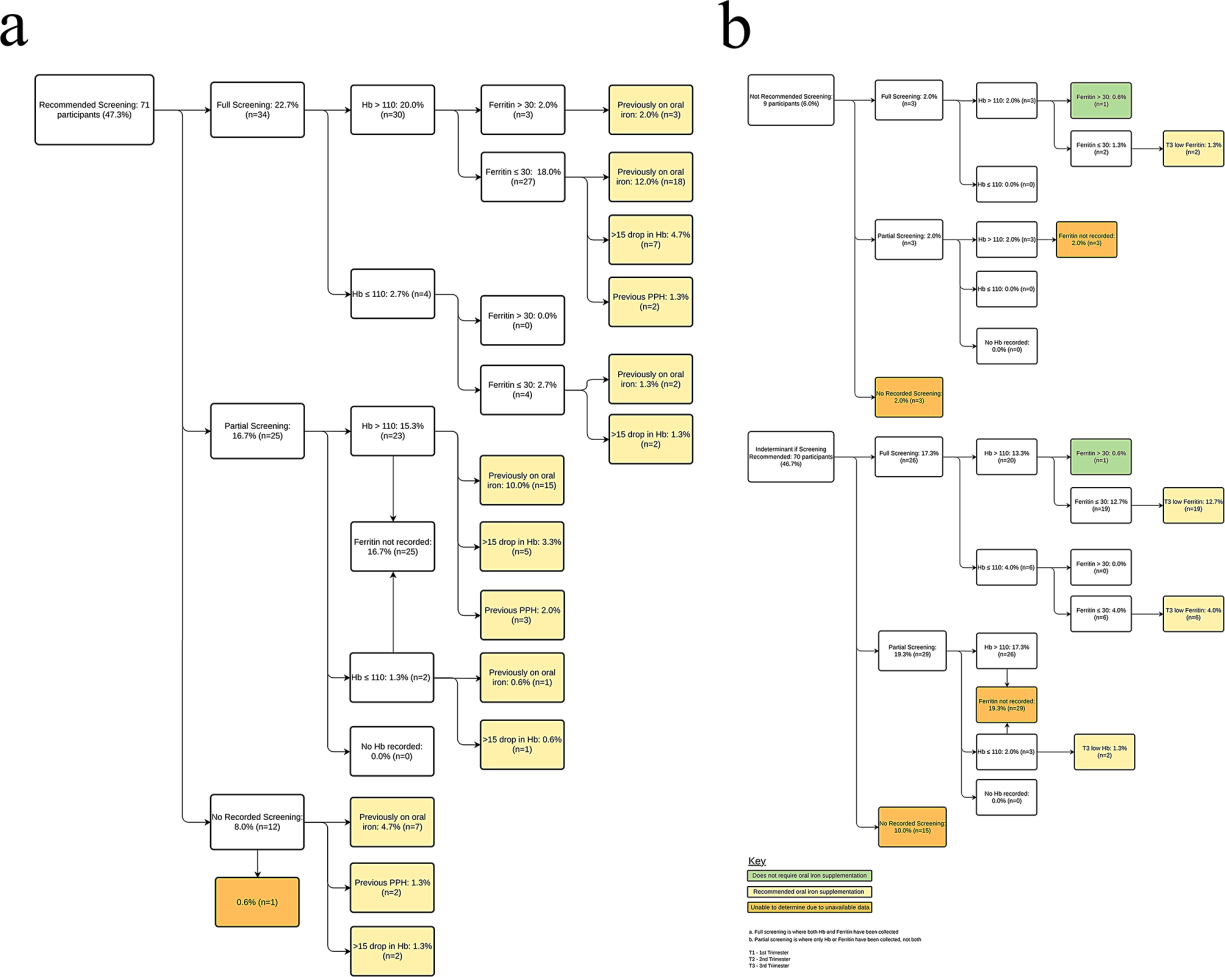
Rates of Iron deficiency in the third trimester for women **A**) recommended for screening and **B**) not recommended or indeterminate for screening

Figures 1 and 2, and 3 delineate anaemia screening rates, blood test completion, and test results by trimesters (T1, T2, and T3) at this hospital. Figure 1 displays screening rates and results, with recommendations for iron supplementation based on RCG. Recommendations were informed by ferritin levels, enabling 33.3% of participants to receive tailored advice in the first trimester. Of these, approximately one-third were advised oral iron supplements due to low ferritin levels. However, only 8.7% of pregnant women recommended for T1 screening were tested, and 27.4% lacked records of recommended screening, despite a quarter being advised supplementation.

Figures 1 and 2 consist of data from all participants, as full screening was recommended for every pregnant woman. Figure 2 delineates second-trimester oral iron supplement recommendations, guided by the ARCG, ferritin levels, Hb change from the first trimester, and previous risk factors like PPH history. Consequently, 53.3% of participants received informed recommendations, with 88.8% advised oral iron supplements, predominantly due to low ferritin levels.

In T2, 30.7% of pregnant women were screened for iron levels. In T3, not all participants underwent anaemia screening. Figure 3 depicts iron supplement prescriptions for 2 of 9 women. It delineates third-trimester oral iron supplement recommendations based on ferritin levels, Hb change, PPH history, and prior iron advice. 66.6% received informed recommendations, with 98.0% prescribed oral iron, primarily due to low ferritin levels during pregnancy.

### Treatment: iron supplementation (oral and intravenous)

**Fig. 4:**
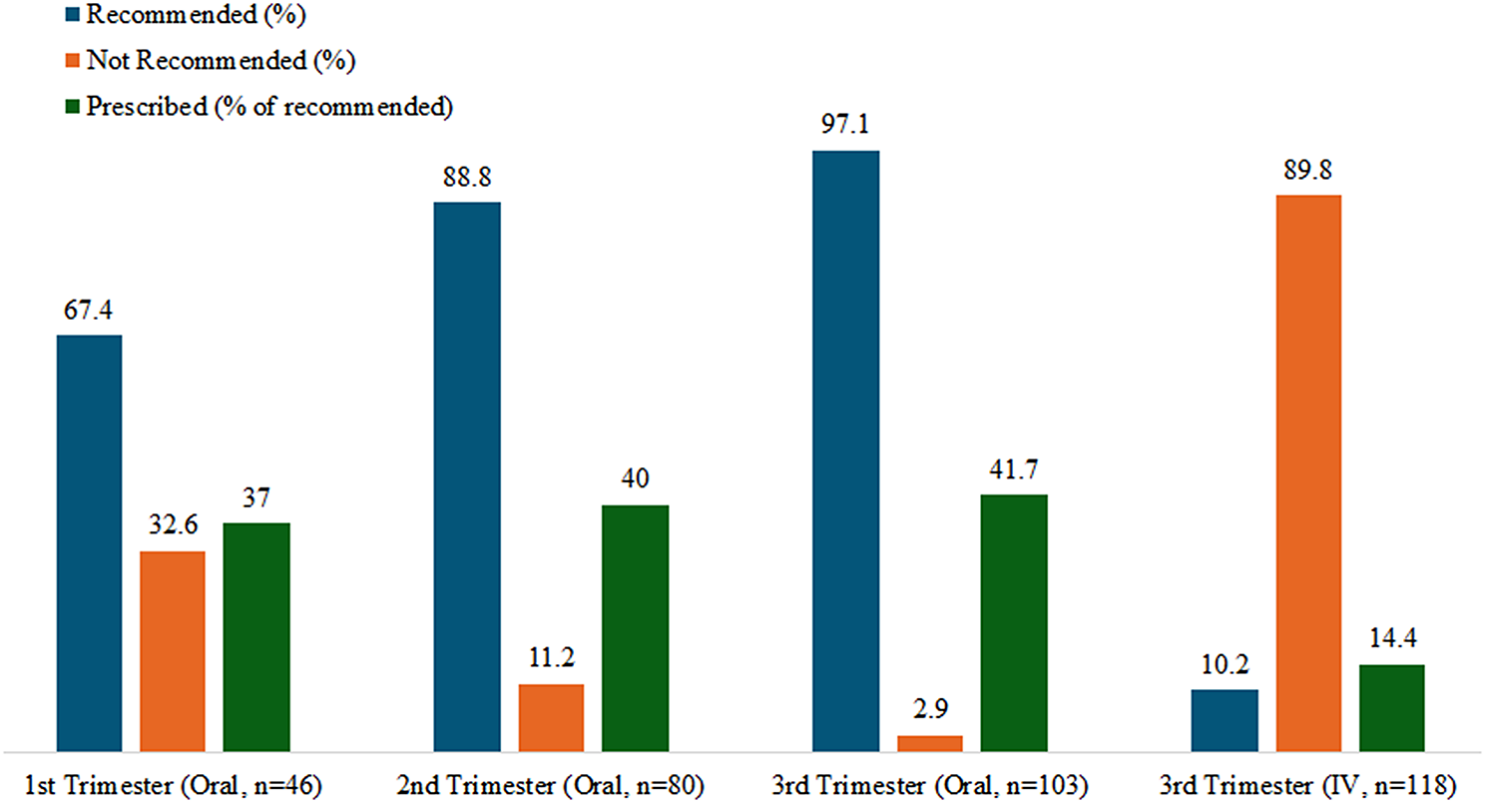
Percentage of women recommended iron supplementation vs. prescription rates

Figure 4 illustrates iron supplementation recommendations versus prescription rates. In the first trimester, 67.4% were advised oral iron due to low ferritin or Hb, > 15 g/L Hb drop, or previous PPH. However, only 37% were prescribed iron. In the third trimester, less than half of the recommended women received prescriptions. IV iron was recommended in 10.2% but prescribed in about 14.4% of cases.

Figure 4 shows iron prescription rates compared to recommended guidelines. 42% received prescribed oral iron, while 6.0% either didn’t require it based on guidelines or lacked documented screening. Additionally, 9.3% received intravenous infusions, despite guidelines suggesting otherwise or lacking screening. In the third trimester, 10.2% were recommended intravenous iron due to non-response, intolerance, or non-compliance with oral therapy, with 58.3% completing the infusion.

### Compliance with treatments

Out of 51 women prescribed oral iron during pregnancy, only 7.8% (*n* = 4) had documented records of medication tolerance and compliance. Among these, 3 reported non-compliance, 2 forgot to take the medication, and 1 discontinued use due to constipation, while another reported constipation but continued using the supplement. None reported immediate adverse effects, and all completed their infusion successfully.

### Postpartum complications

PPH occurred in 40 women (26.7%), with 85% (*n* = 34) during vaginal deliveries. Of the 84 women (56.0%) tested for postpartum Hb levels, 44.0% (*n* = 37) had Hb levels ≤ 110. Among these, 54.0% (*n* = 20) experienced PPH, with 64.9% having antenatal iron deficiency.

## Discussion

Anaemia during pregnancy is a prevalent concern globally, with substantial implications for maternal and foetal health. In this study, we aimed to assess the rate of anaemia screening among pregnant women in regional NSW and evaluate adherence to recommended screening and treatment protocols. The study identified several key findings which to the best of our knowledge, is the first study to assess the rates of rural maternal iron deficiency anaemia as well as compliance to maternal guidelines in rural NSW. This study found an extremely high prevalence of iron deficiency, but only 16.5% were classified as having iron deficiency anaemia during their pregnancy, which was lower than the reported rates of anaemia of up to 25% in pregnant women by the World Health Organisation (WHO). We found no significant relationship between the rates of anaemia and any of the demographic variables, a finding that could be related to the reduced sample size of the study, as several guidelines, including the ARCL guidelines [25], highlighted some of the participant factors assessed in this study, as contributing to low iron. The high prevalence of iron deficiency may be attributed in part to the reported women with unplanned pregnancies in this study, who may already have suboptimal nutritional status before conception which increases their risk of experiencing iron deficiency anaemia [26, 27].

Despite the GPs, midwives, and specialist involvement in this maternity service, the general compliance to screening and treatment guidelines based on the ARCG [25] was very poor across the three trimesters. Screening for full blood counts was seldom conducted during pregnancy, with compliance rates as low as 29.3% in T1 and only reaching 47.9% in T3. Also, screening for Hb levels, but not the ferritin was commonly performed in this study group. Although Hb is a good measure of the current blood status, it is not very predictive of future blood supply. Ferritin levels, on the other hand, can determine the iron stores and thus is more relevant for future red blood cell production, which is particularly important for women who may lose large amounts of blood during labour [28]. The importance of ferritin levels is further highlighted by the recommendation of iron supplements in the majority of the participants with low ferritin levels, despite having normal Hb levels (Fig. 1). There was no publicly available data to compare our study results, however, the Australian Institute of Health and Welfare reported that 92% of women in remote areas attended 5 or more antenatal visits [14]. Largely contrasting our study results, it is important to note our gaps in data, which may have skewed our results.

Regarding treatment for iron deficiency anaemia, the guideline recommended that oral iron supplementation be given to 97.1% of pregnant women, but only 42% were reported to have taken it in our study and only a minority of the participants who were prescribed oral iron, had any follow-up documentation about compliance and side effects. Oral iron was the first line treatment for participants who were recommended iron supplementation, which is in line with the ARC guidelines [25] and the side effects of constipation reported in our study strongly agree with a previous report, which showed gastrointestinal adverse effects as a major deterrent from regular use [20]. To increase compliance, clinicians may consider options of iron supplementation with fewer side effects for these women.

Comparing the treatments provided in this study to the recommended guidelines revealed that the lack of documentation for oral iron prescription and follow-up poses a significant problem. In a previous study, authors identified noncompliance rates of up to 33% for oral iron [20]. A potential cause for this lack of documentation may be the ease of access to oral iron as an over-the-counter medication, which does not require formal prescriptions. The study did not document whether this lack of following guidelines and/or documentation was more among GPs, midwives and specialists lack of following guidelines and /or documentation. About the intravenous iron infusion, the rate of use was 1.75 times higher than the recommended rates and was similar to a previous study [29]. This study revealed that in 9.3% of the participants, IV iron was given where ARC guidelines had not recommended it. It is important to note that this recommendation is solely based on discrete blood values, and does not consider clinical judgment, which is crucial for successful medical care. Of the participants who received an intravenous infusion, there were no reported adverse reactions, as all were completed under close hospital observation. These data differ substantially from a previous study, which reported adverse drug reactions in 24% of women who received an intravenous infusion [30].

Although often documented as a cause of and consequence of PPHs, anaemia was not associated with complications during labour but, the proportion of pregnant women with record PPHs (26.7%) exceeded the national rate of 5 to 15%. Furthermore, the fact that about two in five women experienced a significant postpartum drop in Hb (≤ 110 g/L), emphasises the importance of a substantial iron store before birth to ensure mothers can cope, with the addition of a newborn [31].

### Limitations of study

One limitation of the study is the reduced number of data available for study participants. Due to ethical restrictions, data were only collected from hospital databases (PowerChart and e-maternity), resulting in data not being available for 31.3% (*n* = 47) of participants. Potential reasons may have been that these participants received antenatal care (and completed blood tests) outside of BH, with other providers or that they did not complete the recommended screening. Although the convenience sample covered a large range of demographic factors, statistical analysis was unable to conclusively find any correlations between iron deficiency and various participant variables and risk factors. This may be because of the reduced study population. Another limitation related to the data collected within the hospital antenatal department was gaps in crucial participant information, which although not documented, may have been discussed. This specifically involved the lack of documented follow-up with oral iron supplementations mentioned previously. An additional contributing factor to the gaps in screening may be the presence of multiple different national and state-wide maternal guidelines. Some guidelines require more antenatal screening blood tests than others, resulting in varied levels of screening. Limited by study ethics, it was difficult to search outside hospital databases for participants’ blood records, as the values reported in this study may not be an accurate representation of the hospital’s maternity population. This is especially pertinent to women who received antenatal care and screening outside of the hospital, and where patient information was not transferred over. Future studies would be extremely crucial to further investigating the issue of maternal iron deficiency anaemia in the rural setting. These would include an extension on the current study, increasing the convenience sample as well as the access to participant data. In addition, it was uncertain whether the poor compliance to recommended guidelines for screening was due to poor compliance by staff or women or poor documentation. This could be determined by future studies using qualitative interviews. A potential alteration for a future study, which would improve the current study limitations, would be the use of an alternate set of guidelines such as the Australian Department of Health Guidelines, which divide the antenatal blood into two sets rather than three, allowing one to compare changes throughout pregnancy whilst also requiring less blood values so that there would likely be less data gaps then the current study. While general data on the Australian population is available, there is a lack of studies that specifically examine specific sub-population groups with various levels of access to healthcare.

## Conclusion

This study highlights the gaps in care concerning antenatal screening and general documentation can be improved with staff education and awareness of the potential risks of missed cases. The study was unable to assess the concordance with national maternal guidelines due to missing data and it is unclear if the clinic staff have been trained to follow a protocol of screening. However, this study has provided a snapshot into the antenatal care provided rurally. It also highlights necessary information to community health nurses and staff in the maternity wards to improve antenatal care, ensuring patients are accurately screened and treated, thereby preventing future complications. The findings can be used for local consumption to educate clinicians on following a locally acceptable guideline as well as planning more educational activities for the women they look after. We believe that the findings of our study could contribute valuable insights to the existing body of knowledge on maternal healthcare.

### Electronic supplementary material

Below is the link to the electronic supplementary material.

Supplementary Material 1

Supplementary Material 2

Supplementary Material 3

## Data Availability

The dataset supporting the conclusions of this article is included within the article. Data is also available on request from the corresponding author OUL.

## Abbreviations

ARCL: The Australian resuscitation council
Hb: Haemoglobin
ART: Assisted reproductive technology
PPH: Post-partum hypertension
WHO: World health organization
ARCG: Australian red cross guidelines
