# Supplementary figures and images for: Retrospective audit compares screening and treatment of pregnancy-related anaemia in regional New South Wales with Australian guidelines
Source: BMC Pregnancy Childbirth. 2024 Jul 3;24:457. doi: 10.1186/s12884-024-06634-5 (PMC11223411; doi:10.1186/s12884-024-06634-5)

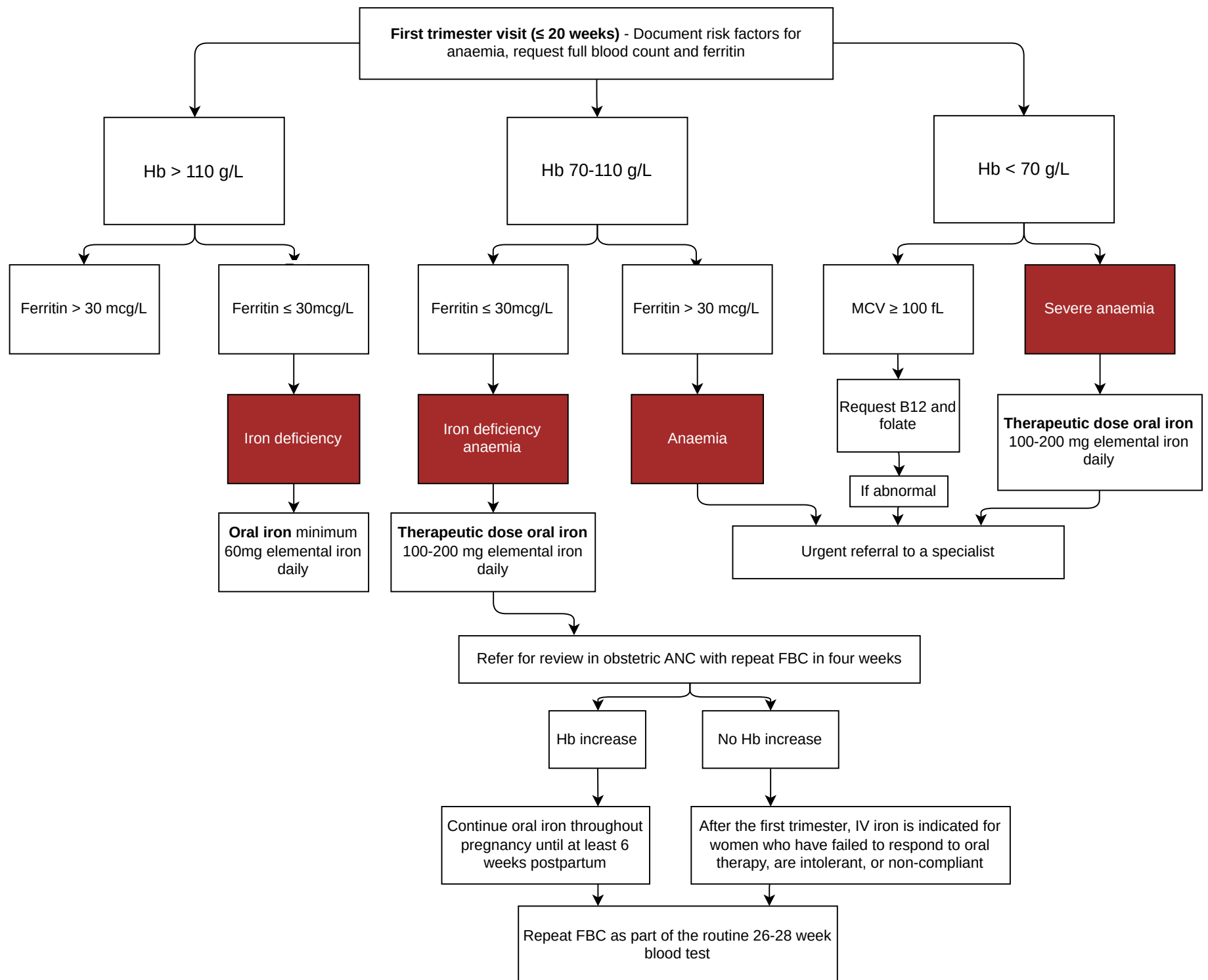

Supplement: Supplementary file 1 — Supplementary Material 1 [file 12884_2024_6634_MOESM1_ESM.pdf]

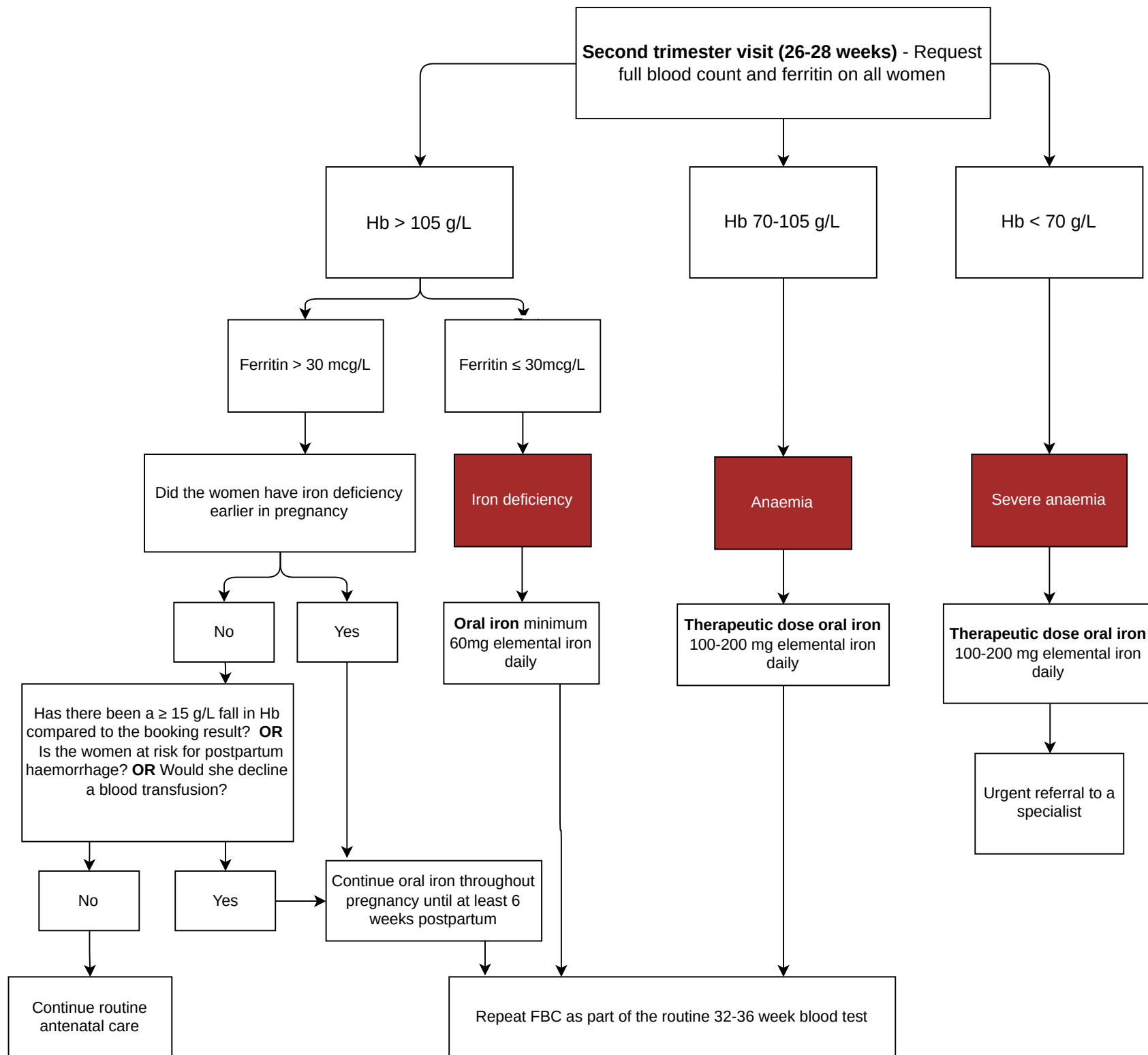

Supplement: Supplementary file 2 — Supplementary Material 2 [file 12884_2024_6634_MOESM2_ESM.pdf]

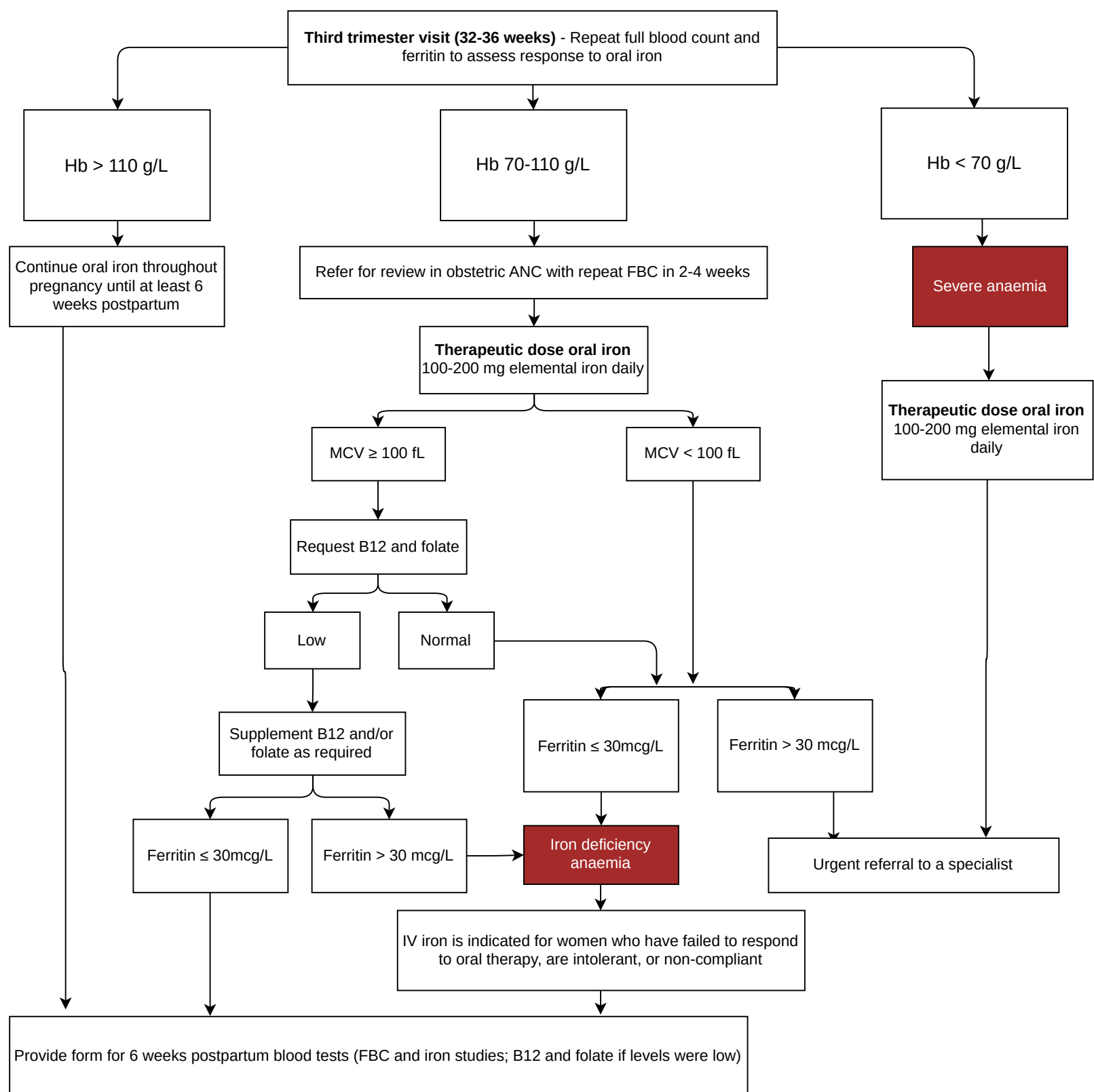

Supplement: Supplementary file 3 — Supplementary Material 3 [file 12884_2024_6634_MOESM3_ESM.pdf]
